# Top-down and bottom-up propagation of disease in the neuronal ceroid lipofuscinoses

**DOI:** 10.3389/fneur.2022.1061363

**Published:** 2022-11-11

**Authors:** John R. Ostergaard, Hemanth R. Nelvagal, Jonathan D. Cooper

**Affiliations:** ^1^Department of Child and Adolescencet, Centre for Rare Diseases, Aarhus, Denmark; ^2^Department of Pediatrics, School of Medicine, Washington University in St Louis, St Louis, MO, United States; ^3^UCL School of Pharmacy, University College London, London, United Kingdom; ^4^Department of Genetics, School of Medicine, Washington University in St Louis, St Louis, MO, United States; ^5^Department of Neurology, School of Medicine, Washington University in St Louis, St Louis, MO, United States

**Keywords:** neuronal ceroid lipofuscinoses, CLN1, CLN3, connectome, disease propagation, neurodegeneration, Brain-first, Body-first

## Abstract

**Background:**

The Neuronal Ceroid Lipofuscinoses (NCLs) may be considered distinct neurodegenerative disorders with separate underlying molecular causes resulting from monogenetic mutations. An alternative hypothesis is to consider the NCLs as related diseases that share lipofuscin pathobiology as the common core feature, but otherwise distinguished by different a) initial anatomic location, and b) disease propagation.

**Methods:**

We have tested this hypothesis by comparing known differences in symptomatology and pathology of the CLN1 phenotype caused by complete loss of *PPT1* function (i.e., the classical infantile form) and of the classical juvenile CLN3 phenotype. These two forms of NCL represent early onset and rapidly progressing vs. late onset and slowly progressing disease modalities respectively.

**Results:**

Despite displaying similar pathological endpoints, the clinical phenotypes and the evidence of imaging and *postmortem* studies reveal strikingly different time courses and distributions of disease propagation. Data from CLN1 disease are indicative of disease propagation from the body, with early effects within the spinal cord and subsequently within the brainstem, the cerebral hemispheres, cerebellum and retina. In contrast, the retina appears to be the most vulnerable organ in CLN3, and the site where pathology is first present. Pathology subsequently is present in the occipital connectome of the CLN3 brain, followed by a top-down propagation in which cerebral and cerebellar atrophy in early adolescence is followed by involvement of the peripheral nerves in later adolescence/early twenties, with the extrapyramidal system also affected during this time course.

**Discussion:**

The propagation of disease in these two NCLs therefore has much in common with the “Brain-first” vs. “Body-first” models of alpha-synuclein propagation in Parkinson's disease. CLN1 disease represents a “Body-first” or bottom-up disease propagation and CLN3 disease having a “Brain-first” and top-down propagation. It is noteworthy that the varied phenotypes of CLN1 disease, whether it starts in infancy (infantile form) or later in childhood (juvenile form), still fit with our proposed hypothesis of a bottom-up disease propagation in CLN1. Likewise, in protracted CLN3 disease, where both cognitive and motor declines are delayed, the initial manifestations of disease are also seen in the outer retinal layers, i.e., identical to classical Juvenile NCL disease.

## Introduction

The neuronal ceroid lipofuscinoses (NCLs), commonly known as Batten disease, are a group of monogenic inherited neurodegenerative disorders that mostly present in the first decade of life (1). Pathologically, these disorders share the common hallmark of intra-lysosomal accumulation of autofluorescent material, called ceroid and lipofuscin, which contains a complex mixture of proteins and lipids (2). These disorders also share a broadly similar clinical presentation characterized by visual failure, epilepsy, and a progressive decline in cognitive and motor abilities. However, these disorders also show marked variation, most notably in the age of onset, rate of disease progression, and initial symptomatology (1). The NCLs were originally classified into four groups according to their clinical onset as infantile, late infantile, juvenile, and adult forms. To date, NCL-disease causing mutations have been revealed in up to 13 different genes: *PPT1, TPP1, DNAJC5, CLN3, CLN5, CLN6, MFSD8, CLN8, CTSD, GRN, ATP13A2, CTSF*, and *KCDT7* (3, 4), with some manifesting as infantile, late-infantile, juvenile, and adult forms, and even a congenital form. The new NCL nomenclature classifies both the defective gene and the age at disease onset and these disorders are now described as “CLN diseases,” CLN1-8, and CLN10-14 (3). The most common forms of NCL are the “classical” infantile type, now called CLN1 disease and caused by mutations in *PPT1*, “classical” late-infantile form called CLN2 disease and due to mutations *TPP1*, and the “juvenile NCL,” or CLN3 disease caused by mutations in *CLN3*. Variant late-infantile forms include CLN5 (*CLN5*), CLN6 (*CLN6*), CLN7 (*MFDS8*), and CLN8 (*CLN8*) which are rarer than CLN1-3, but occur regularly, whereas the CLN10-CLN14 diseases are only reported occasionally.

In many respects, the NCLs are distinct disorders with separate underlying molecular causes, but with a similar pathological endpoint of autofluorescent storage material accumulation and pronounced neuron loss (2). An alternative approach to understand the NCLs would be to consider them as a group of related neurodegenerative diseases characterized by lipofuscin pathobiology as the core feature, but with two defining characteristics used to distinguish each form: (1) the anatomical site of where disease becomes evident (2) the progressive propagation and distribution of disease related to structural and functional dysconnectivity similar to what is seen in other neurodegenerative diseases such as Parkinson's disease (5, 6). So far, investigation of the brain connectome in human NCLs is limited to Juvenile NCL (CLN3), where Roine and coworkers have demonstrated significant global and local network alterations that correlated with the disease severity and in areas related to the symptomatology (7, 8). In the present study, we present and discuss the known differences in symptomatology and pathology between the classical CLN1 phenotype caused by complete loss of *PPT1* gene function (i.e., the classical infantile form) and the classical juvenile CLN3 phenotype. This has allowed us not only to compare and contrast the early onset and rapid progression (typified by CLN1) vs. late onset and slow progression (CLN3) disease modalities, but these are also two of the most common and best documented disease courses among the NCL diseases, both in patients as well as in animal models of NCLs. Using such an approach we conclude that we can broadly classify these two subtypes of NCL into diseases that either display a “Top-down” (CLN3) or “Bottom-up” (classical CLN1) propagation of disease, similar to the recently proposed “Brain-first” vs. “Body-first” model of Parkinson's disease (9, 10). Further, we show that depending on the genotype-phenotype relationship in CLN1 disease, the varied occurrence of age of onset and progression of CLN1 disease phenotypes, whether they be infantile or juvenile, still fits with our proposed hypothesis.

## Search strategy and selection criteria

We searched PubMed for articles written in the English language and published between Jan 1, 1970, and September, 2022, using the search terms “ceroid”, “lipofuscin” “Batten”, “infantile NCL”, “late-infantile NCL”, “juvenile NCL”, “Kufs”, “NCL”, “CLN”, “PPT1”, “TPP1”, “CLN1”, “CLN2”, “CLN3”, “CLN4”, “CLN5”, “CLN6”, “CLN7”, “CLN8”, “CLN10”, “CLN11”, “CLN12”, “CLN13”, and “CLN14”. The final reference list was generated on the basis of relevance to the topic covered.

## Clinical phenotypes

The clinical presentation of most cases of NCL can be variable. This may be due to multiple factors such as (a) effects of different mutations in the same gene (3) that affects genotype-phenotype correlations, (b) how far disease has already progressed at first examination, and (c) the overall rarity of these diseases. Nonetheless, the classical presentations of the most common forms of NCL, and their most common mutations show a consistent pattern of presentation detailed here.

In patients with CLN1 disease (classical infantile NCL), developmental milestones are often within normal ranges until the age of 8–10 months (11). Commonly, the first symptom is mental stagnation starting around one year of age followed by cessation of motor development, as well as irritability and a disturbed sleep cycle that occur around the same time or slightly earlier (12). Thereafter, muscular hypotonia, truncal and limb ataxia, and a severely affected gait function appear. During the third year of life, the disease reaches a so-called “burnt-out” stage (11), where the electroencephalographic recordings (EEGs) show only little cortical activity, the children are rather unresponsive having a permanently increased flexor tone in all limbs, very brisk tendon reflexes and only few or no voluntary movements (11). Epileptic seizure frequency is initially high but tends to decrease in the later stages of disease (1) and all cases are fatal, usually between 7–12 years of age (11, 12).

In contrast, the first symptom in the CLN3 disease or classical juvenile NCL (age of onset 5–7 years) is a rapidly progressive loss of vision, followed by cognitive decline, behavior changes, and progressive epileptic seizures (1). Initially, the CLN3 child seems to walk normally, but recent studies have shown that the walking speed is significantly reduced already at the time of diagnosis (13). At adolescence, extrapyramidal symptoms (rigidity, bradykinesia, slow steps with flexion in hips and knees, and shuffling gait) occur. These extrapyramidal symptoms lead to severe impairments of spontaneous movement and in the late teens the use of a wheelchair becomes necessary (14). At this stage of disease progression, the peripheral motor nerves are involved (15) and the deep tendon reflexes disappear. The patients are usually bedridden at 20–25 years of age; death occurs in the third decade of life, and in some cases even later (1). In CLN3 disease, seizure frequency is initially low, with moderate worsening during later stages of disease (16), unlike CLN1 disease (1).

Retinal degeneration occurs in both subtypes, leading to attenuation of electroretinography (ERG) amplitude. In the classical juvenile CLN3 disease phenotype, ERG already shows significantly reduced rod and cone-response amplitude at the time of diagnosis (5–7 years of age). The responses are abolished three–four years later when the affected children are still mobile and retain their speech, whereas in the classical infantile CLN1 disease, ERG first becomes undetectable at the age of 3 years, i.e., when the children have reached their “burnt-out” stage having only few or none voluntary movements (11). In CLN3, optical coherence tomography (OCT) revealed the first sign of retinal degeneration as a marked thinning of the outer retinal layers followed by a centripetal secondary inner retinal degeneration (17). This suggests an important role of *CLN3* in outer retinal homeostasis, and recently the retinal pigment epithelium (RPE) cells, that both act as a selective barrier to and a vegetative regulator of the photoreceptor layer, have been designated as key targets in CLN3 retinal disease propagation (18). In contrast, although human CLN1 *postmortem* histopathology studies revealed reduced cell numbers in all retinal layers, the peripheral areas showed a significantly better preservation (19), indicating a more centrifugal pattern of disease propagation within the retina.

## Imaging

During the disease course of the classical infantile CLN1 disease, magnetic resonance imaging (MRI) demonstrates a generalized and increasing atrophy of all brain components (20–22). Atrophy begins earliest and proceeds fastest in the cerebrum (about one year of age), with the thalamus lagging slightly behind, whereas atrophy is seen later in the cerebellum (about 2–2½ years of age) and the brainstem (3 years of age). Early bilateral anterior frontal, posterior temporo-parietal and occipital hypoperfusion is observed using single photon emission computed tomography (SPECT) (21). Volume studies show a reduction in cerebellar perfusion in the later stages of disease. However, the perfusion of deep gray matter structures (basal ganglia and thalamus), although atrophic on MRI, is often well preserved up to the terminal stage (20–22).

Conventional magnetic resonance imaging (MRI) of patients with the classical juvenile CLN3 phenotype reveals progressive cerebral, cerebellar, and hippocampal atrophy, decreased gray matter volume in the dorsomedial part of the thalami, and decreased white matter volume in the corona radiata (23–25). However, this cerebral atrophy is not noted until around 9 years and occurs mainly beyond the age of 14 years, beginning in the occipital cortex (24). Thinning of the frontal and parietal cortices occurs later, as do atrophic changes of the brainstem, whereas cerebellar atrophy is seen only around 19 years of age (24). Diffusion-weighted imaging in CLN3 patients revealed significant impairment in the white matter microstructure and brain connectivity networks before the age of ten years, which is substantially earlier than can be demonstrated using conventional MRI (7, 8). Functional studies (PET and SPECT) in CLN3 patients between 15–27 years of age demonstrated thalamic, nigrostriatal and striatal dysfunction as well (26, 27). Investigation of motor conduction velocity in the deep peroneal nerve of CLN3 patients shows a pronounced slowing only in the older (> 15 years of age) and more severe, bedridden cases (15).

## *Postmortem* or autopsy findings

In CLN1 disease, *postmortem* histological studies (28, 29) show an almost complete loss of cortical neurons and secondary loss of axons and myelin sheaths in the white matter (28, 29). The cerebellar cortex shows total loss of granule cells and the cerebellar white matter is almost totally devoid of axons and myelin sheaths (28). Throughout the brain stem and spinal cord, a secondary degeneration of the pyramidal tracts is seen whereas the primary motor nuclei of the brain stem, neurons in the posterior columns, spinocerebellar tracts, the anterior horn cells of the spinal cord, spinal nerve roots or sciatic nerves themselves appear comparatively well preserved (29). In CLN3 disease, *postmortem* investigations have shown symmetrical atrophy of the cerebral hemispheres, cerebellum, and brain stem (30). Histological examination revealed that these changes are seen to be diffusely distributed throughout the layers in all gyri of the cortex, in the cerebellum, the brain stem, and in the spinal cord, with peripheral nerves showing loss of axons and myelin sheaths as well (30). Neurogenic atrophy is also observed in the skeletal muscles, where relatively little autofluorescent storage material is present (30). Thus, at the end stage of the disease, i.e., *postmortem* studies, a marked difference is seen especially within the spinal cord, the peripheral motor nerves and the musculoskeletal system.

In addition, CLN1 and CLN3 exhibit a markedly different propagation of storage material accumulation outside the central nervous system. In CLN3, pronounced deposition of ceroid lipopigment has been shown to occur in the sinus node of the heart, its autonomic nerve supply, as well as in the atrioventricular node and in the bundle of His, and to a lesser extent inside the cardiomyocytes (31). Accordingly, an increasing frequency of conduction disorders including sinus arrest is seen in these patients in their late teens, along with left ventricular hypertrophy in the early 20s (32). In contrast, cardiac involvement is very seldom reported in CLN1 patients and they have no readily apparent cardiac problems during their lifetime except for an increased risk of hypothermia and bradycardia during anesthesia (33).

In CLN1 disease patients, lipofuscin deposits are present in the autonomic ganglion cells of the submucosal and myenteric plexuses and other visceral ganglia (12). Similarly, presence of cellular inclusions has been described in autonomous ganglion cells and other cells in a CLN1 rectal mucosal biopsy specimen as early as 3 months of age (34), i.e., before any signs of illness have occurred. In contrast, in CLN3 patients, lipofuscin inclusions within the autonomous nervous cells of the intestinal wall have been reported only in *postmortem* studies (30), and in diagnostic (*in vivo*) rectal biopsies within smooth muscle cells (35). These variations in lipofuscin deposits also apply to skin biopsies where inclusions are seen almost exclusively in sweat glands among CLN3 patients, whereas in CLN1 patients, the inclusions are seen in a variety of dermal cells (36). Similar inclusions have been reported in skin specimens from autopsy studies of a terminated 20 week old CLN1 fetus (37).

## Discussion

Despite having relatively similar pathological endpoints of retinal, cerebral, and cerebellar atrophy, the documented clinical phenotypes, imaging and *postmortem* studies of CLN1 and CLN3 disease display some strikingly different features in how these diseases progress. CLN3 disease displays a “top-down” propagation of disease related atrophy—starting with abolishment of ERG in the last half of the first decade, followed by cerebral and cerebellar atrophy in early adolescence and loss of postural tone and affection of the peripheral nerves in late adolescents/early twenties, with the extrapyramidal system affected during this time course. In CLN1, lipopigment storage is seen in autonomous ganglion cells and other cells of the rectal mucosa as early as 3 months of age, and in skin cells during the fetal period, i.e., in tissues with a considerable distance from the CNS, and that at a time when there are no clinical manifestations of CNS disease. As the cerebral manifestations start at the earliest at the end of the first year of life, disease progression in CLN1 appears to takes place with a contrasting “bottom-up” propagation.

The distinct patterns of disease propagation in these two forms of NCL and their accompanying clinical manifestations shares many similarities with the “Brain-first” vs. “Body-first” and the alpha-synuclein Origin and Connectome (SOC) models of Parkinson's disease (9, 10). In the context of the NCLs, the “Body-first” progression is comparable to a faster progression of disease represented by CLN1 disease. In contrast, a “Brain-first” progression having a slower onset can be likened to CLN3 disease. This raises the question of whether there is evidence for CLN3 disease pathology arising in the CNS itself, and if so, which cell population is the most vulnerable? Similarly, does CLN1 pathology originate outside the brain, perhaps even in the enteric nervous system as proposed in Parkinson's disease (9, 10), and does this hypothesis also fit other CLN1 subtypes?

There are several indications that the retina is the primary location of CLN3 disease and from where the disease propagates. First of all, visual impairment is the first presenting symptom in more than 80% of CLN3 patients and compared to early-onset Stargardts disease (STGD1), which is the most common macular degenerative eye disease presenting in the first decade of life (38), the retina in CLN3 disease is affected more extensively and faster, involving both cones and rods, and the optic nerve associated layers (39). Secondly, in protracted CLN3 disease, where the cognitive and motor decline is delayed, the retinal phenotype is identical to the earlier onset phenotype of the classical Juvenile NCL disease (40). Thirdly, the presence of a non-syndromic retinal disease in patients with certain mutations in CLN3 showing retinal degeneration without additional progression of CLN3 disease (41–43) further supports that the retina seems to be the most vulnerable organ to *CLN3* deficiency. Finally, the effects of CLN3 disease are present within parts of the visual system connectome at a very early stage (8) and thinning of the occipital cortex occurs years before cortical atrophy is seen in other parts of the brain (24).

Since lipofucsin deposits in CLN1 disease have been demonstrated in peripheral ganglion and dermal cells significantly earlier than in the CNS (34, 35), histological, clinical, and imaging evidence all suggest a “Body-first” or “Bottom-up” disease progression in this subtype. As with CLN3 disease, the mechanisms of such propagation are yet unclear. Nonetheless, the successive nature of clinical and pathological progression along connected cell populations is suggestive of such a modality. At the same time or even before clinical regression is evident, the CLN1 child becomes very irritable, is difficult to comfort and has a disturbed sleep cycle (12), which may indicate an early involvement of some areas of the brainstem, as do the early appearance of increased flexor tone in the limbs and the brisk tendon reflexes. How the effects of CLN1 disease are propagated or even spread to the brainstem and then to the cerebral hemispheres and cerebellum is far from clear, but may potentially either be through, (1) the afferent sensory nerves, dorsal root ganglia and *via* the spinal interneurons and ascending spinal tracts or (2) through the enteric or peripheral autonomic nervous system spreading to the CNS *via* the vagal nerve and the sympathetic connectome. Since the posterior column, spinocerebellar tract, and the anterior horn cell are described to be relatively well preserved in human CLN1 autopsy studies (29), a route of propagation *via* the vagal nerve and/or the sympathetic connectome, as in Parkinson's disease, seems attractive. However, neither route can conclusively be proven as *in vivo* histological examinations of different areas of the spinal cord and brainstem during the CLN1 disease course have understandably not been attempted in humans and remains to be tested experimentally.

**Figure 1:**
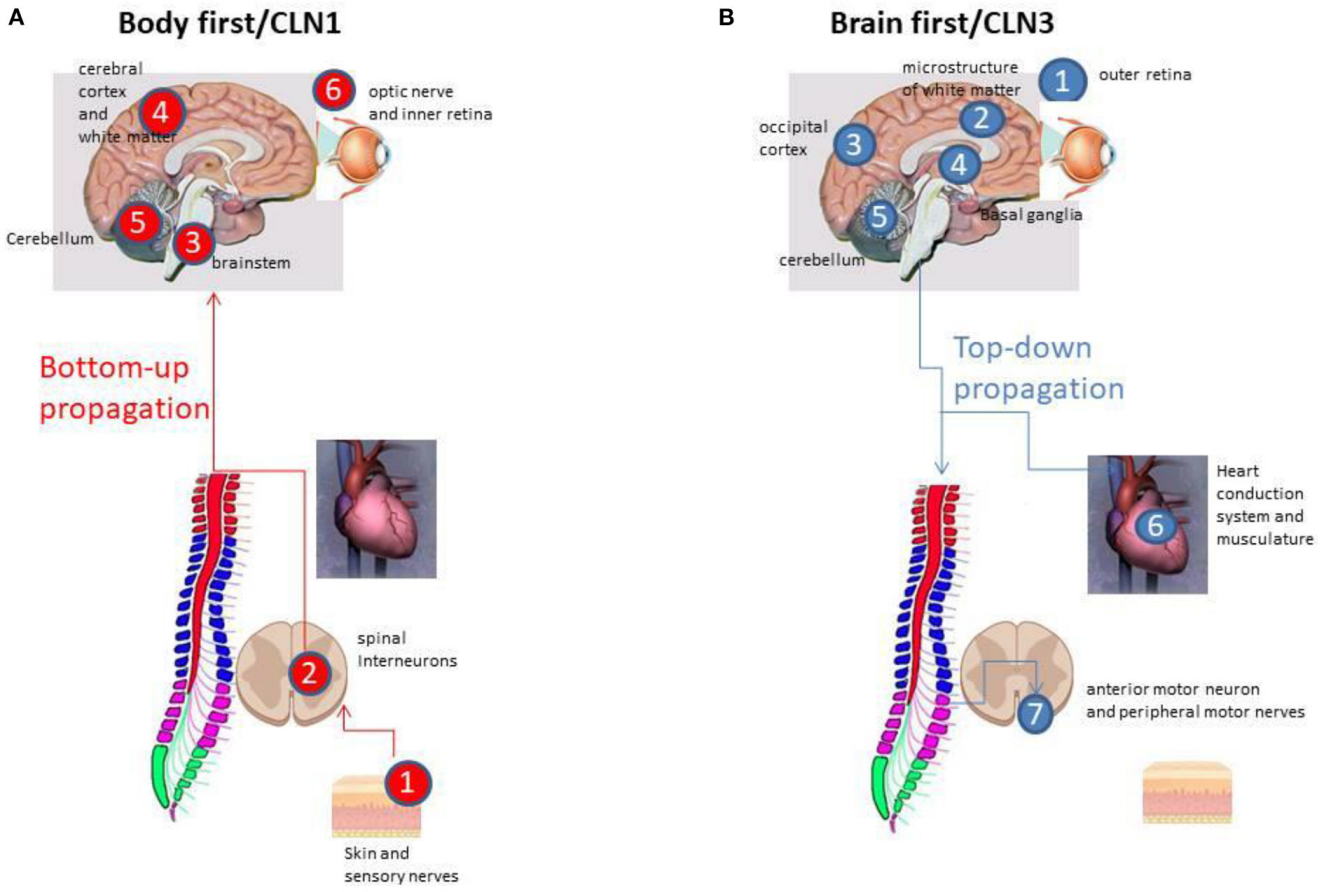
**(A)** The “Bottom-up” propagation in CLN1 disease *via* the peripheral sensory nerves, the dorsal root ganglia and *via* the spinal interneurons to the brainstem (all within the initial 1–1½ years of life), and then into brainstem, the cerebral hemispheres, and, at 2½−3 years of age, affecting the cerebellum and retina. **(B)** The “Top-down” disease propagation in CLN3 starting at the outer retina 5–7 years of age, followed by cerebral and cerebellar atrophy in early and late adolescence, respectively, with the extrapyramidal system affected in between. Affection of the autonomic nerve supply of the heart, the sinus node and the heart conduction system takes place in late adolescence, whereas impact of the peripheral motor nerves occurs in late adolescents/early twenties.

A comprehensive evaluation of the nature and timing of early CLN1 disease pathology in the spinal cord of the Ppt1-deficient (*Ppt1*^−^*/*^−^) mouse has recently been reported (43). *Pp1*^−^*/*^−^ mice recapitulate most human phenotypes including a shortened lifespan, visual defects, epileptic seizures and gait defects during disease progression (43). *Ppt1*^−^*/*^−^ mice also display pathological changes characteristic of the human disorder (44), including accumulation of autofluorescent storage material, and glial activation that precedes the pronounced neuronal loss that occurs in the brain, cerebellum and retina (45, 46). Analysis of the progression of pathology in the spinal cord (43) revealed a very early impact of disease upon the spinal cord, a full quarter of their total lifespan earlier than in any other part of the central nervous system. Glial activation started as early as one month of age, and was followed by a series of pathological events that subsequently included astrocytosis and, at 2 months of age, a selective loss of interneurons in the ventral part of the spinal cord. This all occurred far earlier than similar events within the cerebrum, cerebellum and the motor neurons of the spinal cord (43). Interneurons in the ventral part of the spinal cord are critical for controlling the speed and coordination of locomotion (47, 48), and onset of pathological deterioration of spinal interneurons in these mice was accompanied by a significantly altered gait phenotype (43). This involved altered speed and coordination that is fully consistent with gait deterioration in a child with a well known CLN1 mutation, and it is plausible that spinal interneuron pathology may significantly contribute to an abnormal gait phenotype in CLN1 (43). Simultaneously, in the dorsal horn of the spinal cord, an increased expression of Substance-P and calcitonin gene-related peptide (CGRP) was observed (49). Substance-P and CGRP are neuropeptides associated with the propagation and maintenance of hyperalgesia. This finding may help to explain the restlessness, irritability and sleep disturbances that are reported early in disease course in ~90% of CLN1 cases (12). These data are therefore suggestive of the pathological propagation in the classical CLN1 disease from the periphery, *via* the spinal interneurons, trough ascending spinal tracts, to the brainstem, and then into the cerebral hemispheres, cerebellum and retina (Figure 1). An alternate pathway *via* the enteric autonomic nerves to the brain stem could also potentially occur, but it is apparent that the spinal cord is affected early in CLN1 disease.

In humans, CLN1 disease is caused by various pathogenic variants in the CLN1 gene and shows more than the “classical infantile NCL” phenotype described above. The term infantile CLN1, late infantile CLN1, juvenile CLN1, and adult CLN1 are used for phenotype classification based on age at onset. From a clinical point of view, the time-wise counterpart to the classical juvenile CLN3 disease (JNCL), i.e., the juvenile onset CLN1 type, starts between the ages of 5 and 10 years, and is characterized by a cognitive decline as the initial symptom, followed by motor decline and seizures (7–17 years), whereas vision loss occurs rather late, only after 10–14 years of age (50, 51). In addition, in juvenile CLN1 disease, there is no parkinsonism as is present in JNCL; instead spasticity is seen (1). Thus, although the age of onset is similar, the clinical manifestations and the route of progression between Juvenile CLN1 disease and JNCL (CLN3) are quite opposite, and instead similar to those seen in the classical infantile NCL disease. This means that the occurrence of different CLN1 disease courses of infantile or juvenile nature actually fits well with the proposed hypothesis and does not contradict it.

The current study has its limitations. Primarily, the precise mechanism *via* which the effects of disease may be potentially transmitted along pathways in any form of NCL remains obscure. Whether this process is actually propagated along axons, or the clinical effects disease just happen to become apparent in anatomically connected structures remains to be properly investigated. Indeed, we are yet to fully understand the patho-mechanisms underlying cell-type specific or regional vulnerability in the CNS arising from mutations in ubiquitously expressed lysosomal proteins. Further, being a novel concept it is yet to be tested experimentally in animal models. This is exacerbated by the majority of the pathological and histological studies of human tissue being from 40–50 years ago in a period when a specific diagnosis was not genetically verified. However, the clinical symptoms used today to describe the classical and genetically verified CLN1 and CLN3 subtypes are identical to the clinical descriptions as they appear in the earlier pathological and histological case series of the infantile and juvenile forms of NCLs from the 1970s and 1980s. Another point of criticism might be the children's life expectancy, which has significantly increased over the recent decades, which may mean that there may have been some later changes occurring in the brain and/or the peripheral nervous system which had not been detected due to an earlier death in archival cases from many years ago. This criticism applies especially for the *postmortem* examinations, but is of minor importance in relation to more recent imaging studies that mainly assess the progressive stages of the diseases. Due to the restricted number of histological studies of the spinal cord and peripheral nerves during NCL disease courses in human cases, we cannot exclude alternate routes of disease propagation such as through the enteric or peripheral autonomic nervous system *via* the vagal nerve, as the bottom-up route in CLN1. However, the difference between propagative routes *via* either the afferent sensory nerves to the spinal cord, and especially its interneurons or *via* the enteric nervous system and/or vagal nerve do not detract from the overall caudo-rostral directionality of clinically observed CLN1 disease propagation. Furthermore, not knowing the precise route or mechanism of disease propagation does not affect the proposed differences between CLN3 and CLN1 as a Top-down vs. Bottom-up model, respectively. Finally, the limitations of the proposed models of disease propagation do not significantly affect our hypothesis that although the NCLs certainly share a similar pathological endpoint of pronounced neuron loss and autofluorescent storage material accumulation, they follow quite different sequences of events that converge to reach their end stage.

The “Brain-first” vs. “Body-first” model fully acknowledges the significant roles played by many other factors, including oxidative stress, mitochondrial dysfunction, and calcium dysregulation as well as glial activation (52). Some of these factors increase the probability that the first lipofuscin aggregation and potentially the lysosomal dysfunction that causes it occurs at a specific anatomical location such as the skin, gut or retina and selective transmissibility factors may promote differences in intra-neuronal and neuron-to-neuron propagation of pathology. Equally, it may simply be that within different neurons it takes a variable time for lysosomal dysfunction to reach a threshold where storage material has accumulated sufficiently to be detectable. However, the evidence that the effects of disease appear to occur sequentially along connected neural pathways is unlikely to be a coincidence. In addition, other factors, likely responsible for a selective neuronal vulnerability, may influence how a neuron responds to the presence of lysosomal dysfunction and may explain why certain neuronal populations undergo severe degeneration and cell death, whereas other neurons are more resistant to the presence of NCL pathology during the course of the disease.

NCL disease research is still hampered by a relatively poor understanding of the underlying disease mechanisms. For example, while the biological actions of the enzyme deficient in CLN1 disease have long been known (2), its normal substrate(s) remains unidentified. Even the precise intracellular location of action for several NCL proteins, including Btn1 (the CLN3 protein homolog in yeast) and how this is affected by mutations, remains unclear (2). While new treatment regimens are emerging in Batten disease, challenging previously held views of disease progression not only shed new light on disease mechanisms, but might also aid the means to intervene therapeutically. The present study demonstrates that there is a great difference in the progression of clinical symptomatology and pathology between two major forms of Batten disease, not only in relation to rate of disease progression, but also which parts of the central or peripheral nervous system are affected first and the extent to which different regions are vulnerable to disease.

This is of great importance when considering how to monitor treatment results during disease course most appropriately, but it also has consequences for which route of administration to choose, as clearly shown in a recent pre-clinical study in *Ppt1*^−^*/*^−^ mice (53). Targeting the spinal cord *via* intrathecal administration of an adeno-associated virus (AAV) gene transfer vector in combination with forebrain-directed gene therapy showed a dramatic and synergistic improvement in motor function with an unprecedented increase in life span when compared to mice in which the same gene therapy was administered at only one of the two sites (53). Such findings raise the important question of how and when the spinal cord, especially the spinal interneurons, is affected in other subtypes of the CLN diseases, which consequently also require different routes of administration to maximize therapeutic efficacy.

The proposed “Top-down” vs. “Bottom-up” hypothesis needs to be evaluated and refined. Mechanistic studies will be needed to reveal its underlying basis not just in mouse models, but also in larger animal models of NCLs like sheep, dogs and pigs. The advent of new genome editing methods such as CRISPR/Cas 9 has enabled the efficient generation of animal models with a disease-causing mutation of choice. Recently, such methods have been used to generate both CLN3 pig (54) and CLN1 sheep (55) models of disease, which more closely recapitulate human disease phenotypes that are not present in mouse models. Characterizing these new larger animal models, including obtaining detailed developmental, behavioral, radiological and pathological landmarks of disease progression will be important to bridge the gap between smaller animal models and our understanding of human disease. In particular, to establish connectome-based biomarkers to track NCL disease progression, predict outcomes and monitor treatment responses in both animals models and human beings will be a great improvement for the understanding and targeting treatment options for the NCLs.

## Data availability statement

The original contributions presented in the study are included in the article/supplementary material, further inquiries can be directed to the corresponding author.

## Author contributions

JO and JC contributed to conception and design of the study. JO, HN, and JC organized the search database and wrote sections of the manuscript. JO wrote the first draft of the manuscript. All authors contributed to manuscript revision, read, and approved the submitted version.

## Conflict of interest

The authors declare that the research was conducted in the absence of any commercial or financial relationships that could be construed as a potential conflict of interest.

## Publisher's note

All claims expressed in this article are solely those of the authors and do not necessarily represent those of their affiliated organizations, or those of the publisher, the editors and the reviewers. Any product that may be evaluated in this article, or claim that may be made by its manufacturer, is not guaranteed or endorsed by the publisher.
